# Supplementary material for: Gender-Specificity of Fatigue and Concerns Related to the COVID-19 Pandemic—A Report on the Polish Population
Source: Int J Environ Res Public Health. 2023 Apr 5;20(7):5407. doi: 10.3390/ijerph20075407 (PMC10094230; doi:10.3390/ijerph20075407)
Supplement: Supplementary file 1 [file ijerph-20-05407-s001.zip › ijerph-2127368-supplementary.pdf]

SUPPLEMENTARY

Table S1 Sociodemographic part

Gender	
Please indicate your gender	woman
	man
Age	
Please indicate your age	<18 years (exclusion from the study)
	18-29 years
	30-39 years
	40-49 years
	50-59 years
	≥60 years
Accommodation	
Please indicate What is your place of residence	countryside
	town ≤ 200 000
	town 200 000 -500 000
	town ≥ 500
Education	
Please indicate what education level do you have	elementary or junior high school
	basic vocational
	secondary or post-secondary
	higher education
Employment	
Please indicate what is your status in the labor market (one answer):	employed (full-time or self-employed)
	student
	unemployed
	pensioner/Retiree
	household leader
Chronic diseases	
Are you currently being treated for a chronic disease (e.g., diabetes hypertension, COPD)?	No
	Yes, 1
	Yes, more than 1
COVID-19 vaccination	
Are you vaccinated against COVID-19?	No
	Yes, not fully (first dose)
	Yes, fully (two doses or single-dose formulation)
Pass the COVID-19	
Have you undergone COVID-19 infection?	Yes
	No
	I do not know

Table S2 Pandemic Fatigue Scale (PFS): Please indicate to which extent you disagree or agree with the following statements:

PFS	1	2	3	4	5	6	7
	Strongly disagree	Disagree	I rather disagree	No opinion	I rather agree	I agree	I strongly agree
I am tired of all the COVID-19 discussions in TV shows, newspapers, and radio programs, etc							
I am sick of hearing about COVID-19							
When friends or family members talk about COVID-19, I try to change the subject because I do not want to talk about it anymore							
I feel strained from following all of the behavioural regulations and recommendations around COVID-19							
I am tired of restraining myself to save those who are most vulnerable to COVID-19							
I am losing my spirit to fight against COVID-19							

Table S3 Subjective Fatigue Symptoms (SFS-6): Please indicate how much are you currently experiencing the following aspects of pandemic fatigue: not at all, to a small extent, moderately, fairy intensively or very intensively

	1	2	3	4	5
	Not at all	To a small extent	Moderately	Fairly intensively	Very intensively
Difficulty in focusing attention and organizing one's own mental activity					
Emotional tension and/or mental discomfort					
Feeling overwhelmed by the need to stay connected online with people while feeling a lack of real social contact					
A perception of over-stimulation associated with the use of technology for online contact (e.g., multiple applications, frequency of online meetings, use of a camera, etc.).					
A sense of lack of energy and/or reduced ability to perform daily tasks					
Sleep problems (drowsiness despite adequate sleep intake or insomnia, disruption of sleep rhythm)					

Table S4 Covid-19 Concerns Checklist (CCC): "How much are you currently afraid of the following situations: not at all, a minor level, moderately, a high level or a very high level"

	1	2	3	4	5
	Not at all	minor level	moderately	high level	very high level
I can be a source of infection for my loved ones					
Losing someone you love					
Health system overload					
Diseases (other than COVID-19) in the pandemic situation					
Unemployment growth					
Price growth					
Financial difficulties due to loss of income					
Economic crisis					
Re-lockdown					
Unpredictability and uncertainty of the course of the pandemic					

Table S5 Differences between respondents age categories in pandemic fatigue (PFS, PFS info, PFS behav), subjective fatigue symptoms (SFS) and CCC

	18-29<br>M (SD)	30-39<br>M (SD)	40-49<br>M (SD)	50-59<br>M (SD)	≥60<br>M (SD)	F	p
PFS	5.02 (1.44)	5.13 (1.53)	4.68 (1.66)	4.35 (1.72)	3.77 (1.64)	33.114	<.001
PFS info	5.14 (1.41)	5.34 (1.44)	4.78 (1.61)	4.55 (1.62)	3.74 (1.49)	28.184	<.001
PFS behav	4.68 (1.43)	4.75 (1.58)	4.37 (1.63)	4.08 (1.66)	3.46 (1.56)	29.572	<.001
SFS	2.77 (1.08)	2.77 (1.12)	2.58 (1.03)	2.38 (1.03)	2.23 (0.95)	13.526	<.001
CCC	3.34 (.64)	3.41 (.82)	3.41 (.73)	3.35 (.84)	3.37 (.69)	.378	.825

Table S6 Differences between respondents according to treatment for chronic diseases in pandemic fatigue (PFS, PFS info, PFS Behav), subjective fatigue symptoms (SFS) and Covid-19 Concerns Checklist (CCC)

	No<br>M (SD)	Yes, 1<br>M (SD)	Yes >1<br>M (SD)	F	p
PFS	4.67 (1.68)	4.39 (1.66)	3.94 (1.69)	11.131	<.001
PFS info	4.87 (1.63)	4.56 (1.56)	4.06 (1.69)	14.201	<.001
PFS behav	4.30 (1.65)	4.11 (1.64)	3.74 (1.62)	6.263	.002
SFS	2.48 (.94)	2.57 (.95)	2.60 (1.03)	1.243	.289
CCC	3.32 (.74)	3.47 (.72)	3.38 (.73)	4.717	.009

Table S7 Differences between respondents according to COVID-19 vaccination in pandemic fatigue (PFS, PFS info, PFS Behav), subjective fatigue symptoms (SFS) and Covid-19 Concerns Checklist (CCC)

	No<br>M (SD)	Yes, 1st dose<br>M (SD)	Yes, full<br>M (SD)	F	p
PFS	5.29 (1.52)	4.67 (1.34)	4.11 (1.66)	66.859	<.001
PFS info	5.47 (1.41)	4.61 (1.31)	4.31 (1.63)	60.555	<.001
PFS behav	4.91 (1.57)	4.55 (1.46)	3.80 (1.58)	56.890	<.001
SFS	2.57 (1.15)	2.94 (0.89)	2.45 (1.01)	7.343	<.001
CCC	3.26 (.82)	3.38 (.80)	3.44 (.68)	6.127	.002

Table S8 Differences between respondents according to pass the COVID-19 in pandemic fatigue (PFS, PFS info, PFS Behav), subjective fatigue symptoms (SFS) and Covid-19 Concerns Checklist (CCC)

	No<br>M (SD)	I do not know<br>M (SD)	Yes<br>M (SD)	F	p
PFS	4.35 (1.58)	4.78 (1.36)	4.39 (1.53)	5.709	.003
PFS info	4.62 (1.67)	5.07 (1.40)	4.54 (1.66)	6.512	.002
PFS behav	4.08 (1.68)	4.48 (1.56)	4.23 (1.61)	4.446	.012
SFS	2.41 (1.08)	2.68 (0.99)	2.73 (0.98)	10.171	<.001
CCC	3.34 (0.74)	3.45 (0.69)	3.45 (0.75)	2.345	.096
